# Supplementary material for: Efficient Somatic Embryogenesis, Regeneration and Acclimatization of Panax ginseng Meyer: True-to-Type Conformity of Plantlets as Confirmed by ISSR Analysis
Source: Plants (Basel). 2023 Mar 10;12(6):1270. doi: 10.3390/plants12061270 (PMC10053578; doi:10.3390/plants12061270)
Supplement: Supplementary file 1 [file plants-12-01270-s001.zip › plants-2240771-supplementary.pdf]

Efficient Somatic Embryogenesis, Regeneration and Acclimatization of *Panax ginseng* Meyer: True-to-Type Conformity of Plantlets as Confirmed by ISSR Analysis

Supplementary Materials

Jung-Woo Lee [1,2], Jang-Uk Kim [1], Kyong-Hwan Bang [1], Nayeong Kwon [1], Young-Chang Kim [3], Ick-Hyun Jo [4] and Young-Doo Park [2,*]

[1] Department of Herbal Crop Research, National Institution of Horticultural and Herbal Science, Rural Development Administration, Eumseong 27709, Republic of Korea
[2] Department of Horticultural Biotechnology, Kyung Hee University, Yongin 17104, Republic of Korea
[3] Research Policy Bureau, Rural Development Administration, Jeonju 54875, Republic of Korea
[4] Department of Crop Science and Biotechnology, Dankook University, Cheonan 31116, Republic of Korea

*Correspondence: ydpark@khu.ac.kr; Tel.: +82-10-3338-9344

Table S1. ISSR primers for evaluating the genetic fidelity of the plants regenerated from somatic embryogenesis of *P. ginseng*

Primer code	Sequence (5′–3′)	Number of amplicon	Amplicon size range (bp)
UBC809	AGA GAG AGA GAG AGA GG	5	370–590
UBC818	CAC ACA CAC ACA CAC AG	1	680
UBC821	GTGTGTGTGTGTGTGTT	1	750
UBC827	ACA CAC ACA CAC ACA CG	2	700–770
UBC868	GAAGAAGAAGAAGAAGAA	4	380–770
UBC878	GGATGGATGGATGGAT	1	680

ISSR, inter-simple sequence repeats

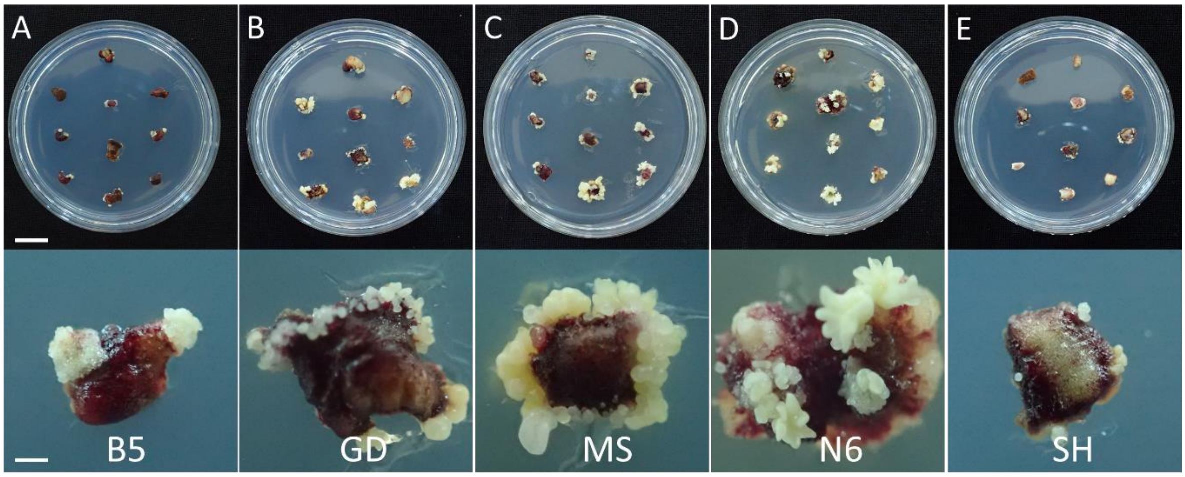

Figure S1. Somatic embryogenesis as influenced by basal media in *P. ginseng* 60 days after inoculation. (**A**) B5, (**B**) GD, (**C**) MS, (**D**) N6, and (**E**) SH. Scale bars, upper 1 cm; bottom 1 mm.

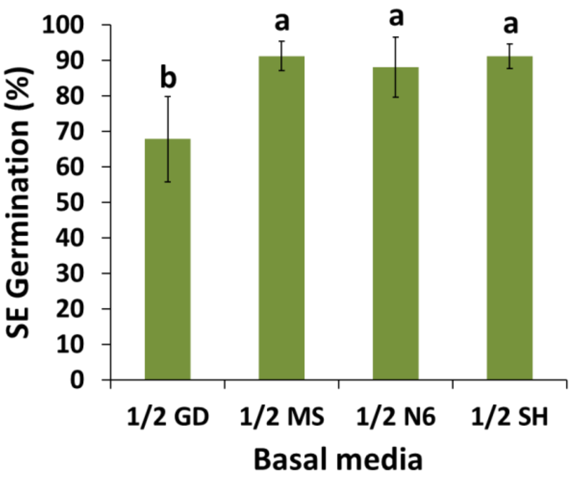

Figure S2. Effect of basal media type in the germination media on SEs germination of *P. ginseng*. Data were recorded 30 days after transfer SEs to germination medium. Values represent the mean ± standard errors of three independent experiments each of which consisted of 10 explants. The non-significant or significant differences were determined by the ANOVA. Different letters within each column represent a significant difference at $p \leq 0.05$ based on Duncan's multiple comparison test. SEs, somatic embryos

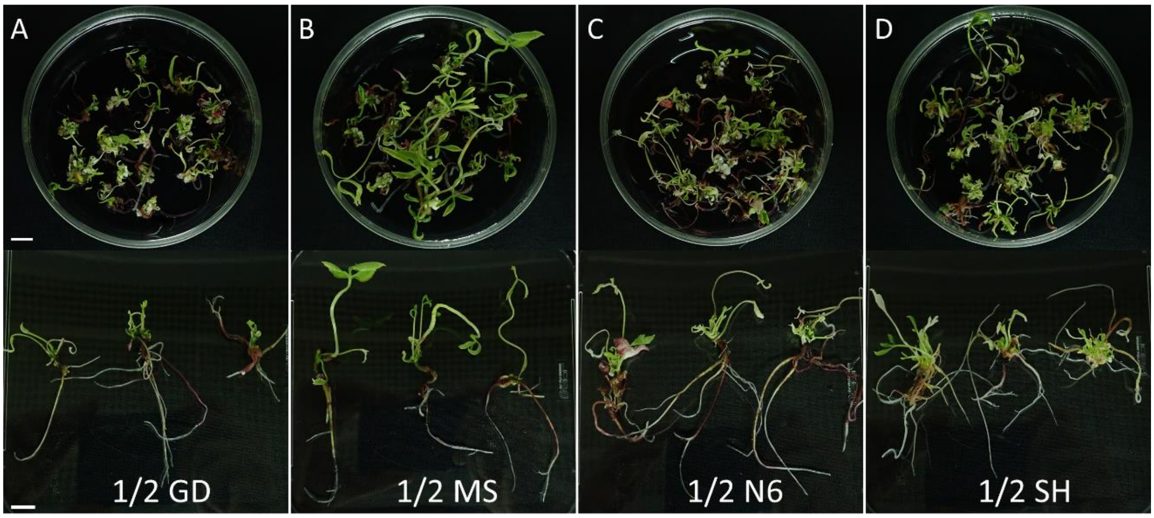

Figure S3. Plant regeneration as influenced by the basal media in the germination medium 60 days after transfer to the elongation medium. (**A**) 1/2 GD, (**B**) 1/2 MS, (**C**) 1/2 N6, and (**D**) 1/2 SH. Scale bars, 1 cm.
